# Practical Realization of Reactive Jamming Attack on Long-Range Wide-Area Network

**DOI:** 10.3390/s25082383

**Published:** 2025-04-09

**Authors:** Josip Šabić, Toni Perković, Dinko Begušić, Petar Šolić

**Affiliations:** Faculty of Electrical Engineering, Mechanical Engineering and Naval Architecture in Split, University of Split, 21000 Split, Croatiapsolic@fesb.hr (P.Š.)

**Keywords:** LoRaWAN, reactive jamming, internet of things (IoT), security, countermeasures

## Abstract

LoRaWANs are increasingly recognized for their vulnerability to various jamming attacks, which can significantly disrupt communication between end nodes and gateways. This paper explores the feasibility of executing reactive jammers upon detecting packet transmission using commercially available equipment based on software-defined radios (SDRs). The proposed approach demonstrates how attackers can exploit packet detection to initiate targeted interference, effectively compromising message integrity. Two distinct experimental setups, one using separate SDRs for reception and transmission, and another leveraging a single SDR for both functions, were used to evaluate attack efficiency, reaction times, and packet loss ratios. Our experiments demonstrate that both scenarios effectively jam LoRaWAN packets across a range of spreading factors and payload sizes. This finding underscores a pressing need for enhanced security measures to maintain reliability and counter sophisticated attacks.

## 1. Introduction

The Internet of Things (IoT) has revolutionized modern information systems by enabling seamless, large-scale connectivity among diverse devices. This ecosystem spans sensors, actuators, and mobile devices that communicate via standard network protocols. While short-range applications frequently employ GHz-based technologies (Wi-Fi, Bluetooth, ZigBee), long-range communications require more energy-efficient solutions, namely Low-Power Wide-Area (LPWA) networks operating in the MHz band [1,2,3,4,5].

LPWA networks are fundamental to the development of smart ecosystems, including urban infrastructure, agriculture, and industrial monitoring. Their capabilities—extended coverage (up to 15 km outdoors), minimal energy consumption, and low data rates—make them ideal for applications such as environmental monitoring, smart lighting, waste management, and precision agriculture [6,7]. LoRa technology, in particular, stands out for its ability to support long-range communication while ensuring extended battery life for IoT devices, facilitating deployments in remote or inaccessible locations [8,9].

Operating within unlicensed industrial, scientific, and medical (ISM) frequency bands, LoRa, SigFox, and NB-IoT networks exhibit distinct characteristics in terms of communication modes, data rates, and cost of deployment. LoRa networks employ chirp spread spectrum (CSS) modulation, while LoRaWANs utilize this radio technology alongside a specific MAC layer to manage network communications. Frequency plans, channel assignments, spreading factors, and payload capacities vary regionally, with the European sub-1 GHz band encompassing 863 to 879 MHz and 433 MHz [10].

However, LoRaWANs’ architecture also presents significant security challenges. The absence of centralized coordination for medium access control (MAC) increases susceptibility to network congestion and malicious interference. In particular, energy depletion attacks (EDAs) threaten battery-powered devices [11], while frame collisions, duty cycle limitations (typically 1% under ETSI regulations), and uncoordinated transmissions amplify the vulnerability of the network [12,13,14,15]. These limitations become more pronounced as network density increases, with future deployments anticipated to involve thousands of devices operating within a single gateway’s range.

One critical threat to LoRaWANs is jamming, where malicious actors transmit disruptive signals to interfere with legitimate communication. This type of attack can undermine essential IoT applications, including alarm systems, fire detection, and environmental monitoring [16,17]. Jamming can target specific channels or spreading factors, with reactive jamming being particularly effective. In this method, attackers identify active transmissions and promptly emit interfering signals, disrupting packet delivery while minimizing their transmission footprint to avoid detection [18].

This paper extends our previous work on reactive jamming attacks on LoRaWANs, originally presented in [19]. Although the previous study demonstrated the feasibility of reactive jamming using two separate SDRs, its effectiveness was limited to only longer packets because of its slow reaction time. This work significantly expands on those findings by introducing much faster reaction times, including a single-device SDR configuration, accompanied by an experimental setup. In contrast, it broadens the analysis to all LoRaWAN scenarios (including fastest packets), with a more comprehensive analysis of reaction times, packet loss ratios, and interference effectiveness across all LoRaWAN spreading factors. Furthermore, we present an analysis of power spectral density, cumulative distribution functions for reaction times, and mitigation strategies to improve LoRaWAN security against reactive jamming attacks. This study explores the feasibility of executing reactive jamming attacks on LoRaWANs using commercially available software-defined radios SDRs (under 1000 EUR). The proposed approach demonstrates how attackers can exploit packet detection to initiate targeted interference, effectively compromising message integrity. Through two distinct experimental setups, one using separate SDRs for reception and transmission and another using a single SDR for both functions, this paper evaluates attack efficiency, reaction times, and packet loss ratios. The results highlight the significant vulnerability of LoRaWANs to low-cost jamming strategies while discussing potential countermeasures to improve network resilience against such threats [20,21,22].

## 2. Related Work—Jamming Attacks on LoRaWANs

### 2.1. LoRaWAN Vulnerability to Jamming

Due to LoRaWANs’ reliance on the ALOHA protocol, which lacks collision-avoidance features, it remains highly susceptible to jamming attacks that disrupt communication between devices and gateways [23,24]. Selective jamming involves targeting specific packets or channels, while reactive jamming adapts its strategy based on the network response to interference, complicating detection and mitigation efforts [22,25].

Moreover, LoRaWAN-specific jamming techniques exploit the unique characteristics of LoRa technology’s chirp spread spectrum modulation. Studies have shown that synchronized jamming can effectively disrupt LoRa communications by overwhelming the gateway with noise, leading to a dramatic decrease in network throughput [25,26]. The inherent design of LoRaWANs, which prioritizes long-range communication over robustness against interference, further exacerbates their vulnerability to such attacks [24,27].

### 2.2. Effectiveness of Different Jamming Methodologies

The effectiveness of various jamming methodologies has been a focal point in recent research. Previous studies report a 56% throughput drop when a LoRaWAN experiences continuous jamming [23,28]. Meanwhile, sweep jamming, a dynamic frequency-varying technique, has been proven especially potent against frequency-hopping systems, presenting an additional threat to LoRaWANs [27,29].

LoRa-specific jamming techniques, such as those that utilize the unique properties of chirp signals, have also been explored. These methods can exploit the physical layer vulnerabilities of a LoRaWAN, effectively disrupting communications without the need for sophisticated equipment [25,27]. The combination of these jamming strategies highlights the urgent need for enhanced security measures within LoRaWANs to mitigate the risks posed by such attacks [24,30].

### 2.3. Real-Time Reactive Jamming on All LoRaWAN Channels

The implementation of real-time reactive jamming on all LoRaWAN channels presents a complex challenge that requires a nuanced understanding of both the jamming mechanisms and the underlying network architecture. A real-time reactive jammer can dynamically adjust its jamming strategy based on the detected activity within the network, maximizing its impact while minimizing the likelihood of detection [31,32].

The technical details of the implementation reveal that SDR technology plays a crucial role in the development of effective jammers. SDR-based jammers can be programmed to monitor multiple channels simultaneously and adapt their jamming signals in real time, providing a significant advantage over traditional hardware-based jammers [33,34]. Low-cost hardware-based jammers, such as those utilizing Arduino platforms, have also been successfully deployed, demonstrating that effective jamming does not necessarily require expensive equipment [31,35].

However, the optimization of jammer strategies is not without limitations and challenges. Processing latency can hinder jammer response, particularly in fast-paced environments where quick adaptations are necessary [32,36]. Furthermore, detection avoidance remains a critical concern, as jammer deployment must be carefully managed to evade countermeasures employed by network operators [25,32].

### 2.4. Feasibility of Real-Time Multichannel Reactive Jamming

The feasibility of implementing multichannel reactive jamming in real time is based on several key requirements. First, monitoring of the wideband spectrum is essential to identify active channels and adapt jamming strategies accordingly [30,33]. This capability allows jammers to target specific frequencies that are currently in use, thereby increasing the effectiveness of their interference.

Parallel processing of multiple signals is another critical requirement for successful multichannel jamming. Using advanced processing techniques, jammers can simultaneously disrupt multiple channels, complicating the network’s ability to maintain communication [25,27]. Furthermore, adaptive jamming techniques that can bypass the frequency-hopping mechanisms employed by LoRaWANs are vital to ensure sustained disruption [29,37].

Demonstrated multichannel jamming architectures have shown promising results in various studies. For example, Šabić et al. [19] utilized SDR-based reactive jamming to effectively target multiple channels, while Perković et al. implemented a low-cost Arduino jammer that successfully disrupted single-channel communications [31,32]. Dossa et al. also explored SDR-based minimal exposure jamming, highlighting the potential for sophisticated jamming strategies that minimize the risk of detection [38]. Table 1 gives comparison techniques of jamming methods in LoRaWAN studies.

## 3. LoRa/LoRaWAN Message Transmission

This section provides an overview of how LoRa technology transmits messages, including key concepts such as chirp spread spectrum (CSS) modulation, spreading factors, bandwidth, and frequency channels. In addition, it elaborates on the LoRaWAN protocol, which is built on LoRa technology [10].

### 3.1. Chirp Spread Spectrum (CSS) Modulation

LoRa technology relies on chirp spread spectrum (CSS) modulation, spreading the signal across a wide bandwidth to enhance interference resilience, an attribute well suited to long-range IoT deployments [39].

### 3.2. Carrier Frequency (CF)

LoRa technology operates in several frequency bands, with the most widely utilized frequencies falling within the sub-GHz range, specifically around 868 MHz in Europe and 915 MHz in North America. The selection of carrier frequency significantly affects both the transmission range and the penetration capabilities of the signal, where lower frequencies offer superior coverage, but at the cost of reduced data rates [39].

### 3.3. Coding Rate (CR)

The coding rate (CR) determines the proportion of redundancy bits included for error correction purposes. By adjusting the coding rate, a trade-off can be achieved between data rate and communication reliability: higher coding rates improve resistance to noise and interference, but result in lower effective data throughput [39].

### 3.4. Spreading Factor (SF)

The spreading factor (SF) in LoRa modulation directly affects both the data rate and the resistance to interference: higher SFs enhance reliability at the cost of slower throughput [40].

### 3.5. Bandwidth (BW)

The bandwidth (BW) configuration in LoRa directly affects both the spectral efficiency and the data transmission rate. A narrower bandwidth offers increased sensitivity and extended range, whereas a broader bandwidth supports higher data rates at the expense of coverage [40].

## 4. LoRaWAN

### 4.1. LoRaWAN Architecture

**Figure 1:**
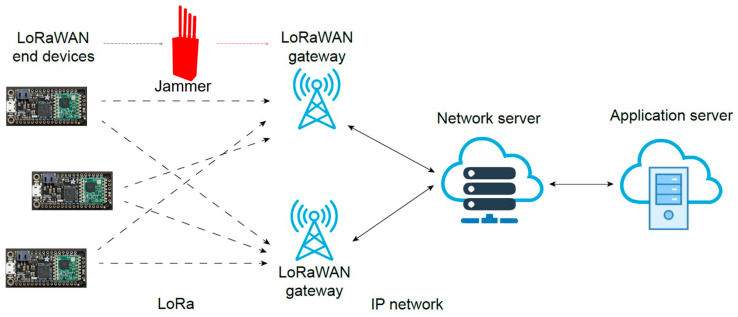
LoRaWAN (Long-Range Wide-Area Network) architecture.

The LoRaWAN architecture consists of end devices, gateways, and a network server that enables communication between devices and applications. The end devices equipped with LoRa transceivers transmit data to gateways, which then send them to the network server for processing before transmitting them to application servers [41]. LoRaWANs employ a star topology (Figure 1), comprising LoRa end devices, one or more gateways, and a centralized network server.

### 4.2. End Devices

LoRaWAN end devices function as sensors or actuators that collect data from the environment or perform specific actions [41]. These devices are optimized for low power consumption, allowing them to operate for extended periods on battery power. LoRaWAN end devices are categorized into three classes: Class A, Class B, and Class C [42]. Class A devices transmit data only when necessary, ensuring energy efficiency. Class B devices are synchronized with periodic time slots to enable scheduled communications. Class C devices maintain continuous reception readiness for immediate downlink communication.

### 4.3. LoRaWAN Packet Structure

**Figure 2:**
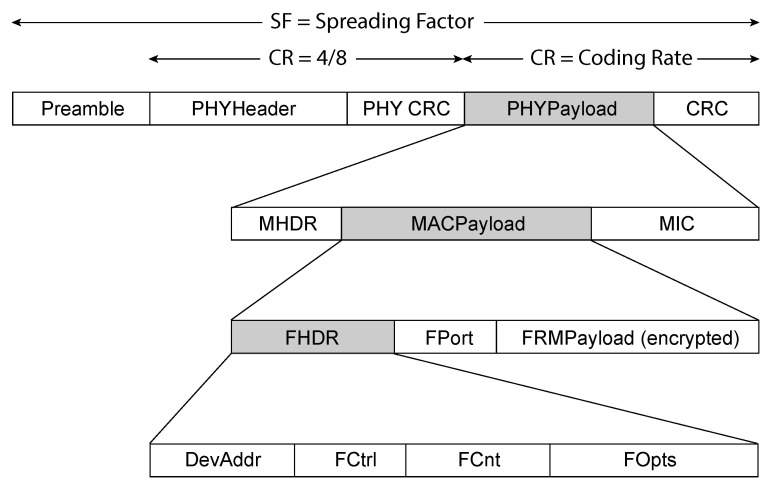
Structure of LoRaWAN (Long-Range Wide-Area Network) packet.

A LoRaWAN packet is structured to ensure reliable and efficient communication between end devices and the network infrastructure. It begins with the physical layer, which includes a preamble for synchronization, a header with metadata, and CRC fields for error detection. The packet also contains a MAC layer, which includes a MAC header (MHDR), a payload (MACPayload), and a Message Integrity Code (MIC) to ensure security. The MACPayload itself is further divided into components such as the Frame Header (FHDR), which provides routing information, the application port (FPort), and the encrypted data payload (FRMPayload), as shown in Figure 2. This structured format allows for secure, error-free communication while facilitating proper routing and device identification [41].

### 4.4. Threat Model

In this study, an active adversary model is considered in the context of LoRaWAN communication between the end nodes and the gateways. The attack scenario involves an adversary positioned between an end device and a gateway, monitoring the initiation of a LoRa transmission. Once the adversary detects the transmission, it determines key parameters such as frequency, spreading factor, and bandwidth. The attacker then transmits a high-power signal on the same channel and spread factor to prevent the legitimate message from reaching the gateway. If the jamming transmission successfully overlaps the original packet before it is complete, the attack effectively disrupts the communication.

To clarify the effectiveness and rationale behind focusing on reactive jamming, Table 2 summarizes key performance criteria across different jamming techniques:

Reactive, broadband, sweep, and selective jamming are based on key performance factors. Broadband, sweep, and reactive jamming all cover the same frequency range but differ in efficiency, detectability, and adaptability. Broadband jamming continuously disrupts all frequencies within the band, making it highly effective but power-inefficient and easy to detect. Reactive jamming listens for active signals across all frequencies and only transmits when necessary, making it more energy-efficient and harder to detect, while still being highly disruptive.

Sweep jamming moves sequentially through frequencies, making it less efficient against LoRaWANs’ dynamic channel selection, as it may not always match an active transmission. Selective jamming, which targets a fixed frequency, is the least effective method, since LoRaWAN devices can transmit on different channels.

Since LoRaWAN devices randomly select transmission channels rather than using synchronized frequency hopping, broadband and reactive jamming are the most effective, while sweep jamming is situational, and selective jamming is the least practical. Reactive jamming, in particular, poses the greatest threat because of its ability to intelligently detect and jam active transmissions with minimal power consumption and lower detectability, making it a critical focus of this study.

## 5. Materials and Methods

This section details the experimental setup and methodology used to implement and evaluate the reactive jamming attack on LoRaWANs .

### 5.1. Hardware and Software Tools Used

- **Hardware**
  Our host computer featured a 12th Gen Intel® Core™ i7-1255U processor (10 cores, 12 threads, 1.7 GHz base, up to 4.7 GHz) and 8 GB of RAM, providing sufficient resources for processing and analyzing incoming radio signals.
  To support the experimental objectives, we employed SDRs, which are versatile communication systems in which traditional hardware components, such as mixers, filters, and modulators, are implemented through software, allowing greater flexibility and adaptability across various communication protocols and frequencies. The setup utilized two notable SDRs: the bladeRF and the HackRF One. Although not as expensive as high-end SDRs, these devices provided sufficient performance for experimental jamming studies.
  The bladeRF operates over a frequency range of 300 MHz to 3.8 GHz, offering up to 28 MHz of instantaneous bandwidth with software-selectable filter options ranging from 1.5 MHz to 28 MHz. It supports arbitrary sample rates of up to 40 MSPS with 12-bit IQ samples. The device is fully bus-powered over USB 3.0, ensuring high-speed data transfer, and includes an external power option via a 5V DC barrel jack.
  The HackRF One covers frequencies from 1 MHz to 6 GHz and supports a maximum sample rate of 20 million samples per second with 8-bit quadrature sampling, enabling versatile transmission and reception capabilities. Together, the host computer and these SDRs provided a robust platform to analyze and manipulate radio signals across a broad spectrum, facilitating advanced wireless communication research and development. A limitation of HackRF One is that it does not support full-duplex operation, which increases reaction time in certain setups.
  For the LoRaWAN components, we used the LILYGO TTGO T-Beam V1.0 as the end node device. This ESP32-based board features integrated LoRa (868/915 MHz) and GPS capabilities, along with Wi-Fi and Bluetooth connectivity. As a gateway, we used the Laird Sentrius RG1xx, an 8-channel LoRaWAN gateway that supports dual-band Wi-Fi and Ethernet connectivity, compatible with various LoRaWAN servers.
- **Software**
  The software implementation of the system was developed using GNU Radio, a powerful open-source software toolkit for building software-defined radios. GNU Radio enabled the modular design of the signal processing flowgraph, allowing seamless integration of various blocks for real-time detection and analysis of LoRaWAN signals. GNU Radio was chosen for its open-source flexibility, allowing real-time signal processing without requiring expensive proprietary tools. In addition, it offers extensive libraries and preexisting modulation/demodulation blocks for LoRa technology, simplifying implementation. It also provides seamless SDR integration with direct support for bladeRF and HackRF One devices.
  A critical component of the setup was the ChirpDetector block, sourced from the gr-LibreLoRa library (https://gitlab.com/jpsimas/librelora, accessed on 1 March 2025). This block was instrumental in identifying LoRa chirps using their unique modulation characteristics, facilitating the extraction of parameters such as spread factor, bandwidth, and normalized frequency. The combination of GNU Radio’s flexibility and the specialized capabilities of the ChirpDetector block ensured efficient and accurate signal processing.

### 5.2. Description of Experimental Setup

The experiments were carried out using two distinct scenarios to evaluate how different hardware configurations affect the efficiency of reactive jamming attacks on LoRaWANs. In this context, the term sink refers to the device or mode that transmits data, which in our case is the SDR operating in transmitter mode. Conversely, the term source refers to the device or mode that receives data, which is the SDR operating in receiver mode. When using a single SDR for both functions, the sink corresponds to the transmitter mode, while the source corresponds to the receiver mode.

**Figure 3:**
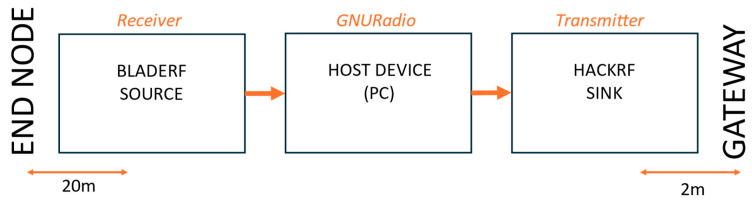
Scenario I block diagram setup.

**Figure 4:**
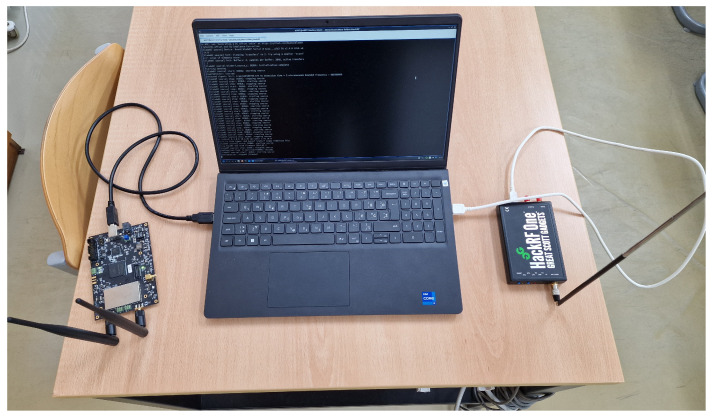
Scenario I setup.

**Figure 5:**
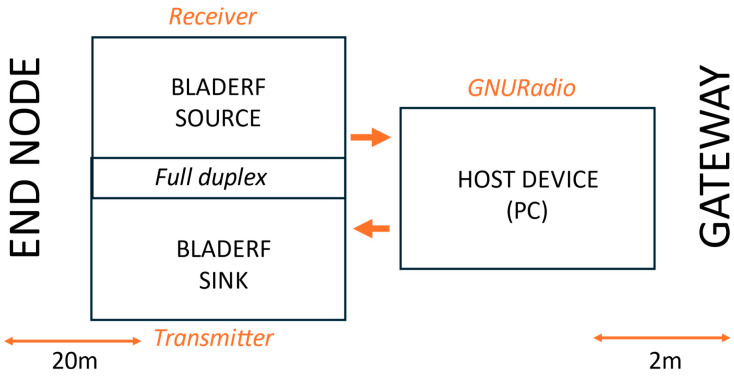
Scenario II block diagram setup.

**Figure 6:**
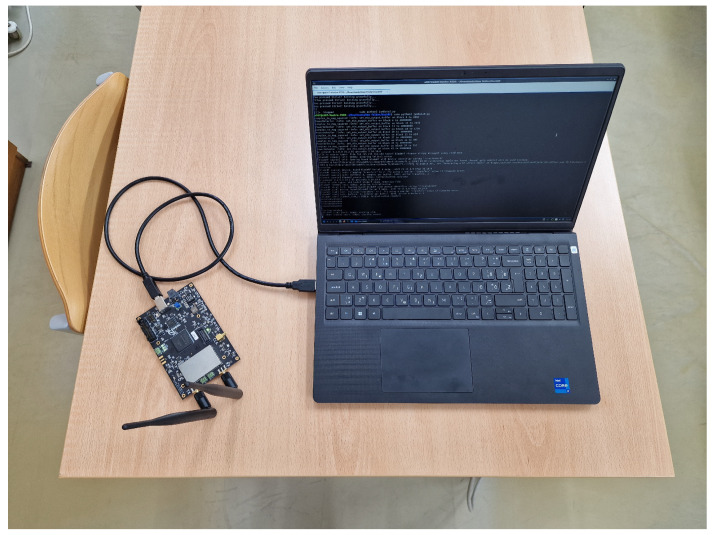
Scenario II setup.

- **Scenario I—bladeRF as receiver, HackRF One as transmitter:**
  Scenario I employs three devices—a bladeRF receiver, a HackRF One transmitter, and a host PC, as illustrated in Figure 3 and shown in Figure 4. The bladeRF monitors the full EU LoRaWAN spectrum and forwards captured signals to the PC for processing to extract key parameters such as the SF, frequency, and BW. Based on the analysis, the HackRF One is used to transmit pre-recorded jamming packets on the detected frequency and SF, effectively interfering with legitimate LoRaWAN communication. This scenario mimics practical attack scenarios with affordable SDRs. We anticipated that this would be a cost-effective setup, offer flexible deployment, allow better control over hardware placement and synchronization testing, and allow decoupling of function for more precise monitoring of the jamming reaction time. However, we anticipated potential disadvantages, including increased latency due to communication between separate SDRs and synchronization challenges with hardware-dependent timing variations.
- **Scenario II—bladeRF as both receiver and transmitter:**
  In this scenario, only two devices are used: the bladeRF and a host device (PC), as illustrated in Figure 5 and shown in Figure 6. The bladeRF SDR operates as both a receiver and a transmitter, monitoring all channels in the EU LoReWAN spectrum. The captured signals are processed on the host device, where the relevant parameters are extracted. Once a target signal is detected, the bladeRF itself transmits the corresponding jamming packets on the detected frequency and SF, eliminating the need for a separate transmitter. This setup ensures a streamlined approach to signal acquisition and jamming within a single device. This scenario was designed to test the hypothesis that a single-device setup could lead to a faster jamming response. We anticipated that the advantages would include lower reaction time by eliminating the need for inter-device communication, improved synchronization by having both reception and transmission within the same SDR, and more efficient jamming due to reduced hardware switching time. However, we also anticipated disadvantages, including a higher processing load on the bladeRF, potentially introducing processing bottlenecks, limited flexibility for experimental variations requiring separate spatial positioning of the receiver and transmitter, and potential RF performance issues due to full-duplex operation in a single SDR.

### 5.3. Step-by-Step Process: Scenario I

**Figure 7:**
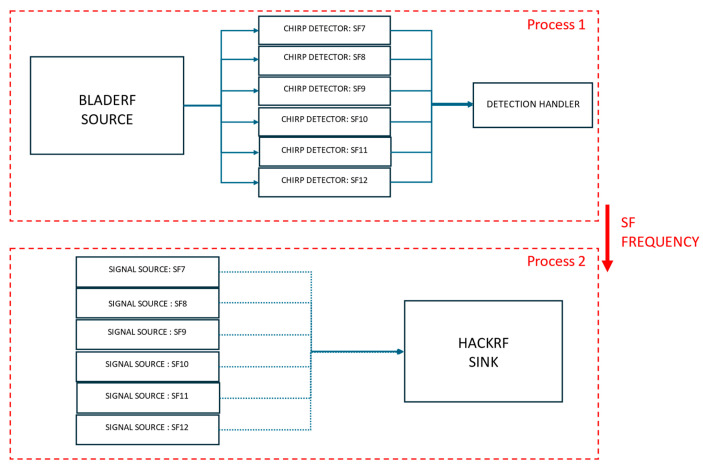
Block diagram of Scenario I.

The step-by-step process for Scenario I is illustrated in Figure 7 and detailed in Algorithm 1.

- **Listening Signal (Process 1)**
  The system employs a structured listening process using an SDR and custom processing blocks to detect LoRaWAN packets with precision. The listening signal operates as a single process, which, through the Python multiprocessing library, shares events and data with a second process responsible for signal jamming. The SDR source, a bladeRF device, is configured to monitor signals within the 868.1 MHz band with a listening bandwidth of 2 MHz. This setup captures incoming radio signals, which are then analyzed through a series of ChirpDetector blocks, each calibrated for an SF ranging from 7 to 12. These detectors identify LoRa chirps and provide key parameters such as the spreading factor, bandwidth, and normalized frequency.
  A custom DetectionHandler block processes events triggered by the ChirpDetector blocks. It calculates the central frequency of the detected signal by using the normalized frequency output from the ChirpDetectors, along with the SDR’s sampling rate and center frequency. Detected frequencies are then aligned to the nearest predefined LoRaWAN channel frequencies, such as 867.1 MHz or 868.3 MHz. When a valid chirp is detected, its parameters, including SF and frequency, are recorded, and a detection flag is raised to initiate subsequent processes, such as signal jamming.
  The SDR source is connected in parallel to all ChirpDetector blocks, with detection events relayed to the DetectionHandler through message ports for real-time processing. This efficient architecture enables accurate detection and characterization of LoRaWAN packets, forming the foundation for advanced signal analysis and interference mechanisms.
- **Jamming Signal (Process 2)**
  The jamming process is implemented using a dedicated SDR and pre-recorded legitimate LoRaWAN packets for each SF. These precomputed signals are stored in memory and serve as inputs for the SDR sink, which is responsible for their transmission.
  When a valid detection is signaled by the detection flag, the system retrieves the detected SF and frequency from the first process. It then dynamically sets the frequency of the SDR sink to match the detected LoRaWAN signal. The system selects the appropriate pre-recorded and pre-loaded signal source corresponding to the detected SF and connects it to the SDR sink. Once the selection is complete, the flowgraph is started, initiating the jamming transmission.
- **Synchronization Between Processes**
  The sensing and jamming processes in the system are designed to run concurrently, leveraging Python’s multiprocessing module to enable parallel execution and efficient synchronization. Multiprocessing provides a way to create separate processes that can execute tasks simultaneously, each with its own memory space. In the context of this system, multiprocessing is used to handle the independent operations of signal detection and jamming while ensuring they remain coordinated.
  Shared variables are a core feature of the multiprocessing framework, allowing real-time communication between processes. In this implementation, shared variables store key parameters such as the detected frequency and SF, ensuring both the sensing and jamming processes have consistent and accurate data. For instance, the detected frequency is updated by the sensing process and then accessed by the jamming process to adjust the transmitter’s center frequency dynamically.
  In addition to shared variables, the system uses event flags to coordinate actions between processes. The detection flag signals the jamming process to start when a valid detection occurs. The jamming flag indicates when the transmitter is actively jamming, ensuring the sensing process pauses to avoid interference. The cooldown flag enforces a mandatory waiting period after jamming, preventing immediate consecutive detections and allowing the system to stabilize.
  By combining multiprocessing’s ability to run parallel tasks with shared variables and event-driven synchronization, the system achieves real-time responsiveness and operational efficiency. This architecture ensures that the detection and jamming processes remain tightly integrated while operating independently, a critical requirement for time-sensitive signal processing tasks.

**Algorithm 1** Reactive Jamming—Scenario I
  1:
**Process 1: Listening (BladeRF)**
  2:**while** *True* **do**  3:    signal ← ReadSignalFromBladeRF (868.1 MHz, 2 MHz bandwidth)  4:    **for** SF in {SF7, SF8, SF9, SF10, SF11, SF12} **do**  5:        **if** ChirpDetector(signal, SF) == *True* **and not** jammingFlag **then**  6:            frequency ← CalculateCenterFrequency(signal)  7:            alignedFrequency ← AlignFrequencyToLoRaWANChannel(frequency)  8:            **if** alignedFrequency is valid **then**  9:                detectedSF ← SF10:                detectedFreq ← alignedFrequency11:                detectionFlag ← *True* {Signal jamming process}12:           **end if**13:        **end if**14:    **end for**15:
**end while**
16:
**Process 2: Jamming (HackRF)**
17:**while** *True* **do**18:    **if** detectionFlag == *True* **then**19:        detectionFlag ← *False*20:        SetHackRFrequency(detectedFreq)21:        jammingPacket ← SelectPreRecordedJammingPacket(detectedSF)22:        TransmitJammingPacket(jammingPacket, HackRF)23:        jammingFlag ← *True*24:        Packet transmission25:        Wait(cooldownPeriod)26:        jammingFlag ← *False*27:    **end if**28:
**end while**



### 5.4. Step-by-Step Process: SCENARIO II

**Figure 8:**
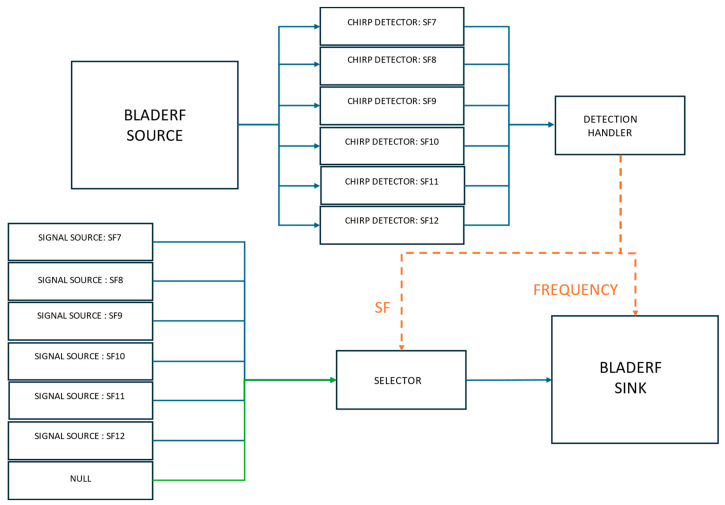
Block diagram of Scenario II.

The step-by-step process for Scenario II is shown in Figure 8 and described in Algorithm 2.

- **Listening Signal**
  The listening process follows the same logic as described in Scenario I, utilizing the bladeRF SDR to capture and analyze LoRaWAN signals with high precision.
- **Jamming Signal**
  The jamming process is integrated within the same flowgraph used for signal detection, ensuring uninterrupted SDR operation. Since the parts of the flowgraph cannot be dynamically turned on or off, a selector block is used to control the jamming mechanism. The selector dynamically switches between a pre-loaded jamming waveform and a zero-filled vector, allowing the bladeRF to transmit interference only when necessary while remaining active at all times.
  Upon detecting a LoRaWAN signal, the detection flag triggers the switching input of the selector block. Initially, the selector block is set to the zero-filled vector (indicating no transmission). Once jamming is required, it dynamically switches to the appropriate jamming waveform for the detected SF. The bladeRF’s transmitter aligns its center frequency to match the detected signal, ensuring accurate interference. After a predefined duration, the selector block is reset to the zero vector, halting jamming while keeping the flowgraph operational.
- **Signal Processing and Synchronization**
  Unlike Scenario I, this implementation operates within a single process, managing sensing and jamming sequentially. This reduces overhead and simplifies synchronization, while maintaining real-time responsiveness.
  Event flags coordinate the system’s state transitions. The detection flag initiates jamming, temporarily pausing chirp detection to prevent interference with the transmitted signal. A cooldown period follows each jamming event, allowing the system to stabilize before resuming normal sensing operations.

**Algorithm 2** Reactive Jamming—Scenario II
  1:**while** *True* **do**  2:    signal ← ReadSignalFromBladeRF (868.1 MHz, 2 MHz bandwidth)  3:    **for** SF in {SF7, SF8, SF9, SF10, SF11, SF12} **do**  4:        **if** ChirpDetector(signal, SF) == *True* **and not** detectionFlag **then**  5:           frequency ← CalculateCenterFrequency(signal)  6:           alignedFrequency ← AlignFrequencyToLoRaWANChannel(frequency)  7:           **if** alignedFrequency is valid **then**  8:               detectionFlag ← *True*  9:               detectedSF ← SF10:               detectedFreq ← alignedFrequency11:               SetBladeRFTransmitterFrequency(detectedFreq)12:               selectorBlock.switchInput ← jammingWaveform[detectedSF] Switch to jamming waveform13:               Wait(jammingDuration)14:               selectorBlock.switchInput ← zeroFilledVector Switch back to zero-filled vector15:               Wait(cooldownPeriod)16:               detectionFlag ← *False*17:          **end if**18:       **end if**19:     **end for**20:
**end while**



## 6. Results

This section presents the experimental results obtained from implementing the reactive jamming attack on LoRaWANs in two distinct scenarios, as described in Section 5. The primary metric for evaluating the attack’s effectiveness is the packet loss ratio (PLR), which indicates the percentage of packets successfully disrupted by the jamming attack. In addition, the reaction time distribution of the jammer is analyzed, highlighting differences in jamming performance between spreading factors.

### 6.1. Metrics Used to Measure Attack Effectiveness

The effectiveness of the reactive jamming attack was evaluated using two key metrics:

- **PLR**: Defined as the percentage of packets transmitted that were successfully jammed and did not reach their intended destination.
- **Reaction Time Distribution**: Analyzed using the cumulative distribution function (CDF), which illustrates the probability that the jammer reacts within a certain time threshold.

These metrics provide insight into the attack performance under different experimental setups and spreading factors.

### 6.2. Experimental Setup and Methodology

Multiple trials were conducted for each experimental condition, covering all SFs and different payload sizes (1, 5, 15, and 20 bytes). Repeating the trials was essential to ensure statistical reliability of the results and to account for hardware-induced variations in reaction times.

- **Ensuring Reproducibility**: Given that SDR-based systems experience slight timing fluctuations due to processing delays, multiple trials helped confirm the consistency of attack effectiveness.
- **Comparing Different Scenarios**: Conducting multiple trials for Scenario I (HackRF One + bladeRF) and Scenario II (bladeRF only) ensured a fair comparison of the two setups.

For each SF, we transmitted and jammed 100 packets in varying payload sizes, and the PLR was measured for each condition. Reaction times were analyzed using the CDF to observe variations between trials. This approach ensured that our conclusions were robust, repeatable, and statistically valid. Although the mean reaction time was used for a general comparison between Scenario I and Scenario II, we relied on CDFs to visualize the probability of the jammer reacting within a specific time, clarifying reaction time differences across spreading factors. The transmitter, receiver, and gateway were kept at a fixed distance to ensure that distance was not a factor in the results, and attention remained focused on synchronization rather than signal power.

To further analyze jamming effectiveness, both power spectral density (PSD) and spectrograms were recorded using an additional HackRF One SDR, which continuously captured raw IQ samples during the jamming process. The PSD was processed using Python 3.10.12-based tools to compute the power distribution over frequency. Similarly, spectrograms were generated to provide a time–frequency representation, illustrating how the jamming signal evolved over time and aligned with LoRaWAN transmissions. These measurements provided insight into reaction timing, interference patterns, and the overall impact of jamming on network performance.

### 6.3. Detailed Experimental Results

#### 6.3.1. Power Spectral Density Analysis (PSD)

**Figure 9:**
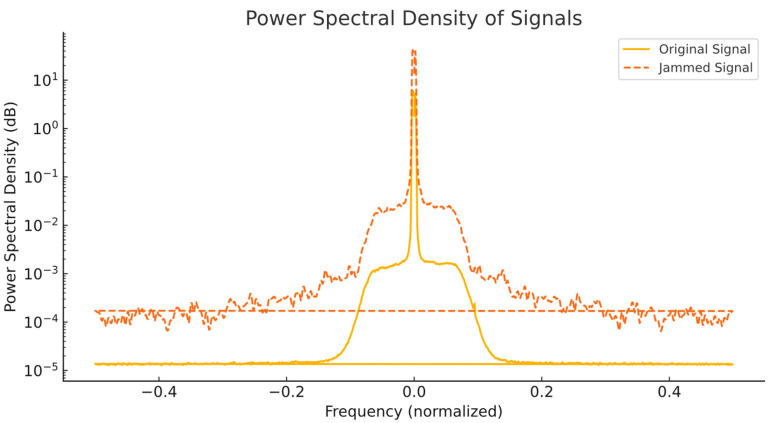
Power spectral density (PSD) of legitimate and jammed signals.

The PSDs of both the legitimate LoRaWAN signal and the jamming signal are shown in Figure 9. This plot highlights the frequency synchronization between the two signals, which is critical for effective jamming.

The PSD plot demonstrates the following:

- The jamming signal aligns closely with the center frequency of the legitimate LoRaWAN signal (normalized to 0), ensuring effective interference.
- The power level of the jamming signal is consistently higher than that of the legitimate signal on overlapping frequencies, effectively overpowering it.

This frequency synchronization is critical for successful jamming as it ensures that key portions of legitimate communication are disrupted. Without proper alignment frequency, the jammer would not effectively interfere with LoRaWAN transmissions.

#### 6.3.2. Scenario I: BladeRF as Receiver, HackRF One as Transmitter

**Figure 10:**
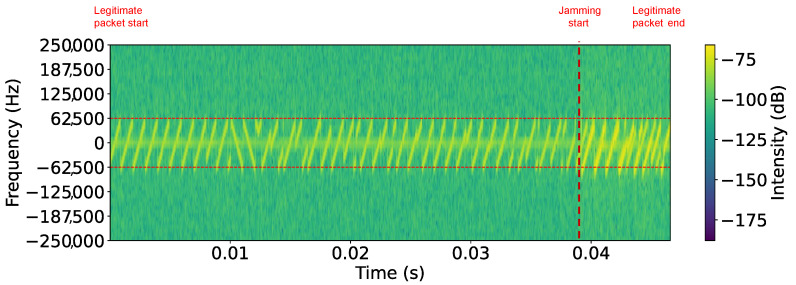
Scenario I: spectrogram of SF7 LoRaWAN jammed packet (1 byte) on 868.1 MHz center frequency.

Figure 10 shows the spectrogram of an SF7 LoRaWAN jammed packet in Scenario I, where the start of the legitimate packet and the onset of jamming are clearly visible and marked. The legitimate packet appears as well-defined, evenly spaced chirps, but once jamming begins, the signal structure becomes distorted and no longer clearly visible, accompanied by a noticeable change in signal power. Table 3 summarizes the PLR achieved in Scenario I. The results indicate a significant impact on LoRaWAN communication, with packet loss 100% achieved across all spreading factors when the payload size was 5, 15, or 20 bytes. However, for a payload size of 1 byte, jamming was slightly less effective in SF7, where packet loss was 50%. Across all trials, payloads of five bytes or more incurred 100% packet loss, highlighting the power of reactive jamming.

**Figure 11:**
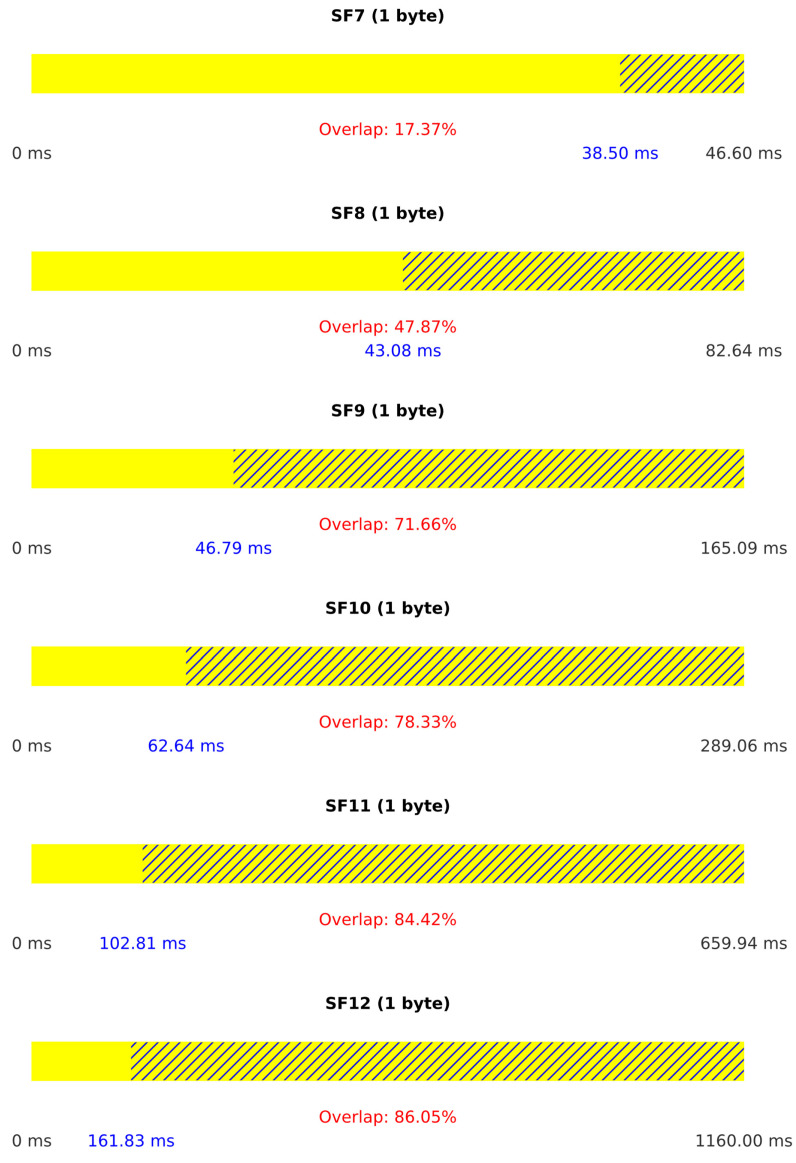
Results for Scenario I.

Figure 11 provides additional insight into the attack performance by illustrating the duration of legitimate packets for each spreading factor and the percentage of overlap between legitimate and jamming packets. Higher overlap percentages indicate more effective jamming.

#### 6.3.3. Scenario II: BladeRF as Both Receiver and Transmitter

**Figure 12:**
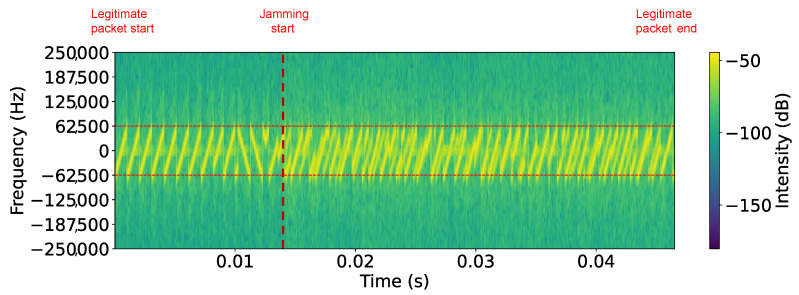
Scenario II: spectrogram of SF7 LoRaWAN jammed packet (1 byte) on 868.1 MHz center frequency.

Figure 12 presents the spectrogram of an SF7 LoRaWAN jammed packet in Scenario II. Like in the Scenario I spectrogram, the start of the legitimate packet and the onset of jamming are clearly marked. The legitimate signal initially appears as well-defined chirps, but once jamming starts, the signal structure becomes distorted. Table 4 presents the PLR for Scenario II, where the reactive jamming attack proved universally effective. Unlike Scenario I, Scenario II achieved a 100% PLR across all SFs and payload sizes, demonstrating the advantage of using the bladeRF for both receiving and transmitting.

**Figure 13:**
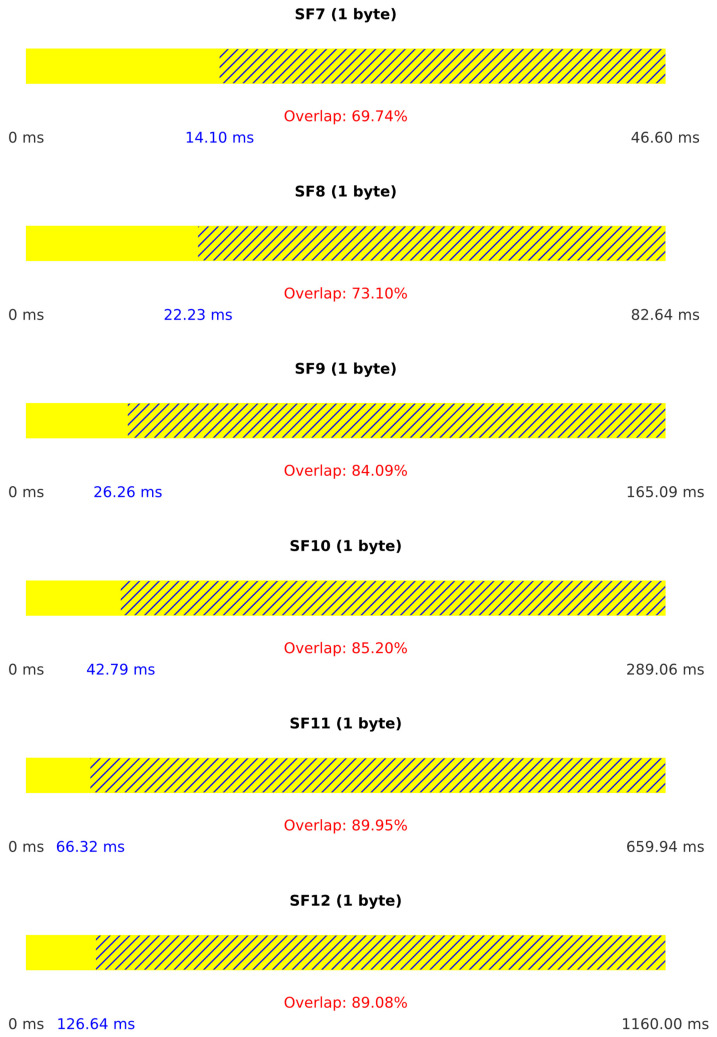
Results for Scenario II.

Figure 13 illustrates the duration of legitimate packets and the percentage of overlap between legitimate and jamming packets. The streamlined single-device operation results in consistently high overlap percentages, explaining the superior jamming effectiveness in this scenario.

**Figure 14:**
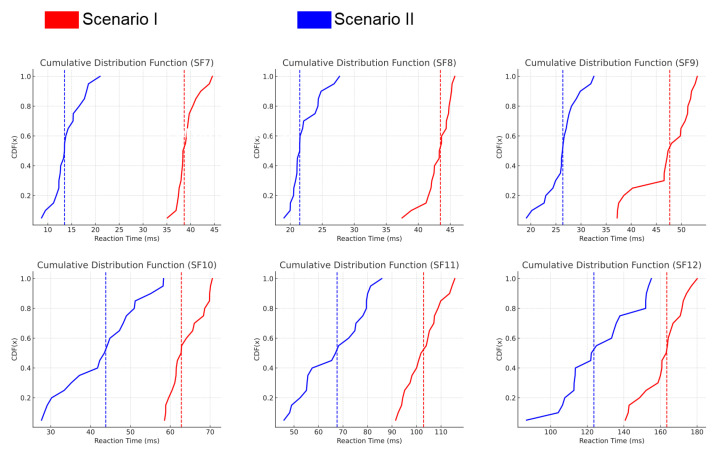
Cumulative distribution function of reaction time for each SF and scenario.

The reaction times of the jamming system in both Scenario I and Scenario II are analyzed using CDF graphs in Figure 14. In both scenarios, reaction times vary based on the spreading factor (SF), with lower SF values resulting in faster reaction times due to shorter symbol durations. In Scenario I, reaction times also show slight variability due to inter-device communication delays, which affects the effectiveness of jamming as shorter reaction times allow greater packet disruption. Similarly, Scenario II exhibits comparable behavior, where faster reaction times enhance the impact of jamming.

To complement these graphical results, Table 5 provides a numerical summary of the mean reaction times and standard deviations for both scenarios.

### 6.4. Limitations

The experimental setup was designed to isolate the impact of the reactive jamming attack on LoRaWANs, and care was taken to keep external parameters consistent in multiple trials. However, the evaluation does have some limitations, such as the transmitter, receiver, and gateway being kept at a fixed distance to ensure that distance was not a factor in the results.

### 6.5. Reactive Jamming Attack on LoRaWAN: A Realistic Scenario

This subsection describes a realistic scenario of our reactive jamming attack targeting a LoRaWAN-based indoor air quality monitoring system. The experiment involved a commercial DL-IAM Indoor Ambiance Monitor device, which transmits environmental data (temperature, humidity, CO_2_, and illuminance) every 10 min over the LoRaWAN.

#### 6.5.1. Experimental Setup

The experimental setup consisted of the following components:

- **DL-IAM Sensor Placement:** The DL-IAM Indoor Ambiance Monitor was placed in office A507.
- **LoRaWAN Gateway Placement:** A Laird LoRaWAN gateway was installed in office A502 to receive sensor transmissions and forward them to The Things Network (TTN).
- **Jammer Placement:** Our reactive jammer was located in office A501, positioned between the sensor and the gateway to effectively disrupt communication.

**Figure 15:**
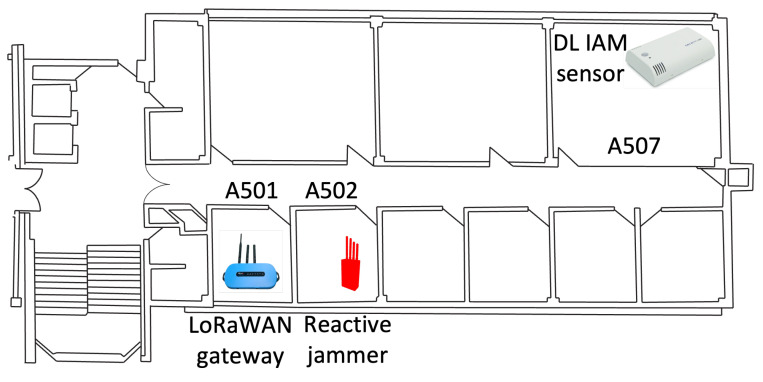
Floor plan showing the strategic placement of the DL-IAM sensor in room A507, the LoRaWAN gateway in room A501, and the reactive jammer in room A502.

**Figure 16:**
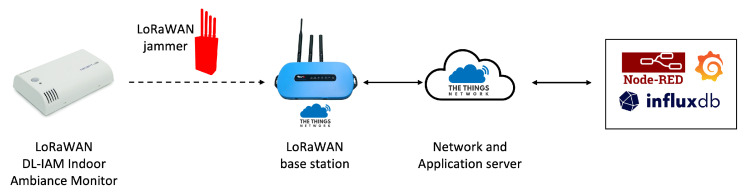
System architecture for the reactive jamming attack.

The physical layout of our experimental setup is illustrated in Figure 15, showing the strategic positioning of all components within the office environment. This arrangement created a realistic scenario where the jammer was positioned to intercept communications between the sensor and the gateway. The overall architecture of the system is depicted in Figure 16.

#### 6.5.2. Attack Implementation and Results

The jammer was active between 15:42 and 16:08, during which it successfully performed a reactive jamming attack on two messages sent by the DL-IAM device. The jammer listened for LoRaWAN transmissions and selectively disrupted them by transmitting interference signals during legitimate communication attempts.

As shown in the floor plan (Figure 15), the physical separation between rooms created a realistic deployment scenario, with the jammer strategically positioned to intercept transmissions between the sensor and the gateway. This layout mimics real-world IoT deployments, where devices may be distributed across different locations within a building.

**Figure 17:**
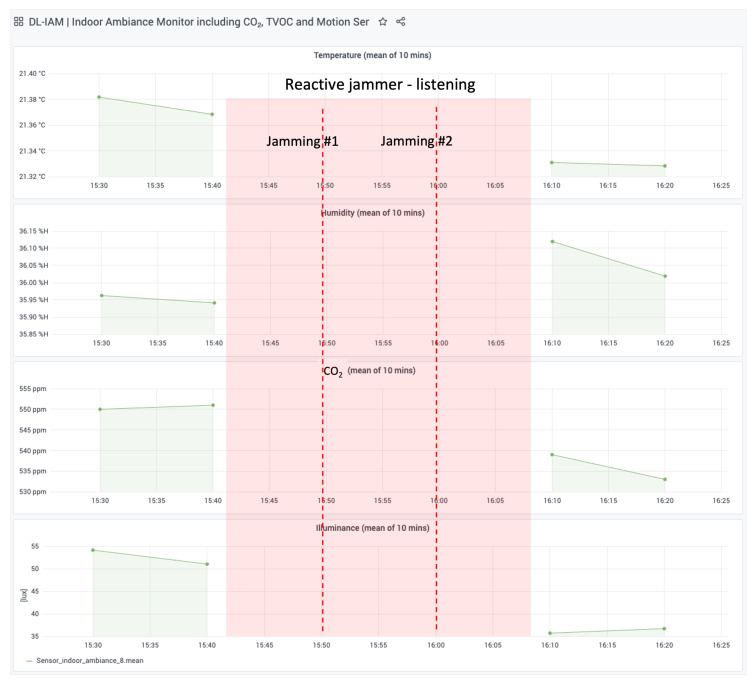
Visualization of reactive jamming results using Grafana. The red-shaded area indicates the jammer’s active period.

The results of the attack are visualized in Figure 17, which shows data collected from TTN and stored in a local InfluxDB database. Key observations include the following:

- **Jamming Events:** Two successful jamming events were recorded at approximately 15:50 and 16:00.
- **Data Interruption:** During the jammer’s active period (highlighted in red), no data were received from the DL-IAM device, confirming the success of the attack.
- **Environmental Parameters:** Temperature, humidity, CO_2_ levels, and illuminance readings show continuity before and after the jamming period, demonstrating normal sensor operation outside the attack window.

This experiment demonstrates how a reactive jammer can selectively disrupt LoRaWAN communications by targeting specific transmissions. The attack demonstrates how a reactive jammer can selectively target LoRaWAN communications by listening for transmission preambles and then transmitting interference signals precisely when legitimate data are being sent. By storing data from TTN in our local InfluxDB and visualizing them through Grafana, we can clearly observe the attack’s impact on network communications while maintaining comprehensive visibility of the entire system’s behavior.

## 7. Discussion

The results of this study demonstrate the significant vulnerability of LoRaWANs to reactive jamming attacks, with both scenarios showing high effectiveness across different spreading factors and payload sizes.

### 7.1. Comparison of Scenarios

Scenario II, using a single bladeRF for both receiving and transmitting, proved to be more effective than Scenario I, which used separate devices for receiving (bladeRF) and transmitting (HackRF One). This difference is particularly notable for SF7 with 1-byte payloads, where Scenario I achieved a packet loss of only 50% compared to 100% in Scenario II.

### 7.2. Jamming Effectiveness and Packet Overlap

Our findings confirm that reactive jamming success is strongly tied to how much the jamming signal overlaps the legitimate packet. In particular, for payloads of five bytes or more, both scenarios consistently yielded a 100% packet loss.

Because partial overlap can invalidate the entire packet, LoRaWANs remain highly vulnerable to targeted interference. Future studies could explore the minimal jamming packet size needed for disruption and whether particular packet fields are more prone to attack.

### 7.3. Reaction Time and Spreading Factors

The CDF graphs show that reaction times vary with spreading factors:

- Lower SFs (e.g., SF7) exhibit faster reaction times due to shorter symbol durations.
- Higher SFs allow for longer reaction times while still achieving successful jamming because of larger duration of the packet.

This variation in reaction times across SFs provides information on the time-sensitive nature of the LoRaWAN packet structure and the critical windows for effective interference.

### 7.4. Legal Considerations and Spectrum Licensing

Our findings highlight LoRaWANs’ technical vulnerabilities to reactive jamming using SDRs. However, practical experiments that involve intentional interference must comply with relevant regulations. Intentional disruption, even within unlicensed ISM bands, is prohibited by regulatory bodies (e.g., FCC, ETSI) and can result in significant penalties. Therefore, interference testing should always be performed in controlled laboratory conditions (such as RF-shielded chambers) or with appropriate regulatory authorization to ensure legal compliance.

### 7.5. Countermeasures

To mitigate the vulnerabilities of LoRaWANs against jamming attacks, several strategies can be implemented.

An effective countermeasure against severe jamming in LoRaWANs is a PHY-layer technique that leverages spatial and temporal diversity. Haque and Saifullah [43] propose JRLoRa, a system where multiple gateways capture the same jammed signal at different timing offsets. These offsets are exploited at the network server to reconstruct the original LoRa packet by subtracting jamming artifacts. The process involves secure sample sharing, synchronization, sample classification (with CNN), and iterative recovery. This approach is fully compatible with COTS devices and significantly improves performance, even when the SNR drops below −35 dBm. Importantly, it does not require changes to the LoRa node’s physical layer.

To mitigate synchronized jamming attacks in LoRaWANs, Hou et al. [44] propose a novel RSS-based decoding technique that enhances resilience at the gateway level. The core idea is to distinguish legitimate LoRa chirps from jamming chirps based on their power differences in the demodulation window. Upon detecting a packet, the receiver uses the preamble to establish a reference power profile. Then, instead of selecting the strongest FFT peak, as in standard decoders, it selects the peak that best matches the expected RSS. This allows the system to recover legitimate symbols even when synchronized jamming chirps are present. This method complements existing time- and frequency-domain countermeasures and achieves significantly higher packet recovery rates without requiring any modification to the end devices.

Integrating the Long-Range Frequency-Hopping Spread Spectrum (LR-FHSS) into the LoRaWAN protocol significantly enhances network capacity and robustness to interference. This technique involves breaking each data packet into smaller pieces and transmitting them over various frequencies, thus reducing the impact of jamming and improving spectral efficiency [45].

Martinez [46] proposes a comprehensive framework for detecting and mitigating jamming in LoRaWANs. The author categorizes countermeasures into proactive, reactive, and detection-based approaches. Among the detection methods, a statistical approach based on the Exponentially Weighted Moving Average (EWMA) is presented, leveraging RSSI and inter-arrival time to flag anomalies. In addition, a recurrent neural network (RNN) is trained to classify normal and jammed states using temporal patterns in traffic features. On the reactive side, the thesis discusses jamming-aware ADR schemes, jamming zone mapping through detection metrics, and the deployment of dedicated detection nodes to monitor spectral activity. These techniques offer layered protection against various jamming strategies without modifying end devices.

Strategies like directive antennas can be employed to focus signal strengths in specific directions, minimizing the risk of jamming by reducing the overall exposure of the signal [47]. By adopting a combination of these strategies, LoRaWANs can enhance their resilience against jamming attacks, ensuring reliable and secure communication for critical applications.

### 7.6. Future Research Directions

1. **Minimal Jamming Packet Size**: Investigate the minimum size of jamming packets (e.g., few chirps) required for successful disruption.
2. **Targeted Jamming**: Analyze which specific parts of LoRaWAN packets are most vulnerable to jamming.
3. **Real-world Deployment Impact**: Assess the effectiveness of the attack in various environmental conditions and network topologies.
4. **Advanced Countermeasures**: Develop and test sophisticated defense mechanisms against reactive jamming.

This study underscores the urgent need for improved security measures in LoRaWANs, especially as IoT deployments continue to expand. The high success rate of jamming attacks, even with partial packet overlap, highlights a significant vulnerability that could potentially disrupt critical IoT applications that rely on LoRaWAN technology.

## 8. Conclusions

We implemented a practical reactive jamming attack on a LoRaWAN using low-cost SDRs (HackRF One and bladeRF) in two experimental configurations. Both approaches severely disrupted LoRaWAN traffic in all spreading factors, reaching 100% packet loss in almost every case. In particular, a single device setup produced faster and more consistent jamming, underscoring the susceptibility of LoRaWANs to time-critical interference.

These findings highlight the inherent vulnerabilities of LoRaWANs when confronted with reactive jamming, particularly given LoRaWANs’ reliance on unlicensed ISM frequency bands and their use of ALOHA-based medium access without robust collision-avoidance mechanisms. Even partial overlap of legitimate and jamming signals was sufficient to prevent successful reception, underscoring the critical nature of precise timing and frequency alignment in attack execution.

Mitigating these vulnerabilities in LoRaWANs requires improvements at both the physical and network layers. Potential defenses include improved spread-spectrum techniques, dynamic channel hopping, or adaptive power and data-rate configurations. Furthermore, real-time jamming detection and proactive response strategies warrant focused research efforts.

As IoT applications relying on LoRaWANs continue to expand into mission-critical domains, addressing these security gaps becomes increasingly urgent. Future research directions should therefore focus on developing and rigorously testing countermeasures, such as early detection and mitigation algorithms, to maintain reliable, secure communication in large-scale IoT deployments.

## Figures and Tables

**Table 1:** Comparison of jamming methods in LoRaWAN studies.

Study	Method Used	Multichannel	All Spreading Factors	Low Cost (<1000 EUR)	Effective in All SF–Freq Combinations	Hardware
**Dossa et al. [38]**	SDR-based minimal exposure jamming	✖	✖	✖	✖	USRP-2920
**Perković et al. [32,36]**	Low-cost Arduino jammer	✖	✖	✔	✖	LoRa module (SX1276)
**Šabić et al. [19]**	SDR-based reactive jamming	✔	✔	✔	✖	HackRF One
**This paper’s Scenario I**	SDR-based reactive jamming	✔	✔	✔	✖/✔	HackRF One
**This paper’s Scenario II**	SDR-based reactive jamming	✔	✔	✔	✔	BladeRF

**Table 2:** Comparison of jamming techniques.

Criteria	Reactive	Broadband	Sweep	Selective
Frequency Coverage				
Power Efficiency				
Implementation Complexity				
Effectiveness against LoRaWAN Channel Selection				
Interference Level (Collateral)				
Detection Probability				
Suitability (Adaptive/Selective use)				
**Legend**				
High	Moderate-High	Moderate-Low	Low	

**Table 3:** Scenario I.

SF/Payload (Bytes)	1	5	15	20
SF7	50%	100%	100%	100%
SF8	100%	100%	100%	100%
SF9	100%	100%	100%	100%
SF10	100%	100%	100%	100%
SF11	100%	100%	100%	100%
SF12	100%	100%	100%	100%

**Table 4:** Scenario II.

SF/Payload (Bytes)	1	5	15	20
SF7	100%	100%	100%	100%
SF8	100%	100%	100%	100%
SF9	100%	100%	100%	100%
SF10	100%	100%	100%	100%
SF11	100%	100%	100%	100%
SF12	100%	100%	100%	100%

**Table 5:** Summary of reaction time statistics (mean ± std. deviation) in ms.

SF	Scenario I	Scenario II
SF7	39.20 ± 2.26	14.10 ± 3.01
SF8	43.08 ± 2.09	22.22 ± 2.27
SF9	46.79 ± 5.41	26.26 ± 3.29
SF10	62.64 ± 7.35	42.79 ± 9.55
SF11	102.81 ± 7.27	66.32 ± 12.55
SF12	161.83 ± 11.23	126.64 ± 19.26

## Data Availability

Data are contained within the article.

## References

[1] Mahdavinejad M.S., Rezvan M., Barekatain M., Adibi P., Barnaghi P.M., Sheth A.P. (2018). Machine learning for Internet of Things data analysis: A survey. arXiv.

[2] Sain M., Kang Y.J., Lee H.J. Survey on security in Internet of Things: State of the art and challenges. Proceedings of the 2017 19th International Conference on Advanced Communication Technology (ICACT).

[3] Sanchez-Iborra R., Cano M.D. (2016). State of the Art in LP-WAN Solutions for Industrial IoT Services. Sensors.

[4] Centenaro M., Vangelista L., Zanella A., Zorzi M. (2016). Long-range communications in unlicensed bands: The rising stars in the IoT and smart city scenarios. IEEE Wirel. Commun..

[5] Mangalvedhe N., Ratasuk R., Ghosh A. NB-IoT deployment study for low power wide area cellular IoT. Proceedings of the 2016 IEEE 27th Annual International Symposium on Personal, Indoor, and Mobile Radio Communications (PIMRC).

[6] Petäjäjärvi J., Mikhaylov K., Pettissalo M., Janhunen J., Iinatti J. (2017). Performance of a low-power wide-area network based on LoRa technology: Doppler robustness, scalability, and coverage. Int. J. Distrib. Sens. Netw..

[7] Adelantado F., Vilajosana X., Tuset-Peiro P., Martinez B., Melia-Segui J., Watteyne T. (2017). Understanding the Limits of LoRaWAN. IEEE Commun. Mag..

[8] Vangelista L., Zanella A., Zorzi M., Atanasovski V., Leon-Garcia A. (2015). Long-Range IoT Technologies: The Dawn of LoRa™. Proceedings of the Future Access Enablers for Ubiquitous and Intelligent Infrastructures: First International Conference, FABULOUS 2015.

[9] Haxhibeqiri J., De Poorter E., Moerman I., Hoebeke J. (2018). A Survey of LoRaWAN for IoT: From Technology to Application. Sensors.

[10] Lora Alliance (2017). LoRaWAN 1.1 Specification. http://lora-alliance.org/lorawan-for-developers.

[11] Mikhaylov K., Fujdiak R., Pouttu A., Miroslav V., Malina L., Mlynek P. Energy Attack in LoRaWAN: Experimental Validation. Proceedings of the 14th International Conference on Availability, Reliability and Security (ARES’19).

[12] Butun I., Pereira N., Gidlund M. (2019). Security Risk Analysis of LoRaWAN and Future Directions. Future Internet.

[13] Yang X., Karampatzakis E., Doerr C., Kuipers F. Security Vulnerabilities in LoRaWAN. Proceedings of the 2018 IEEE/ACM Third International Conference on Internet-of-Things Design and Implementation (IoTDI).

[14] Dönmez T.C., Nigussie E. (2018). Security of LoRaWAN v1. 1 in backward compatibility scenarios. Procedia Comput. Sci..

[15] Butun I., Pereira N., Gidlund M. Analysis of LoRaWAN v1.1 Security: Research Paper. Proceedings of the 4th ACM MobiHoc Workshop on Experiences with the Design and Implementation of Smart Object (SMARTOBJECTS’18).

[16] Aras E., Small N., Ramachandran G.S., Delbruel S., Joosen W., Hughes D. Selective Jamming of LoRaWAN Using Commodity Hardware. Proceedings of the 14th EAI International Conference on Mobile and Ubiquitous Systems: Computing, Networking and Services (MobiQuitous 2017).

[17] Aras E., Ramachandran G.S., Lawrence P., Hughes D. Exploring the Security Vulnerabilities of LoRa. Proceedings of the 2017 3rd IEEE International Conference on Cybernetics (CYBCONF).

[18] Noura H., Hatoum T., Salman O., Yaacoub J.P., Chehab A. (2020). LoRaWAN security survey: Issues, threats and possible mitigation techniques. Internet Things.

[19] Šabić J., Perković T., Šolić P. (2024). Chirp Detection and Signal Transmission: A HackRF Reactive Attack. Proceedings of the 2024 International Conference on Smart Systems and Technologies (SST).

[20] Lee J., Hwang D., Park J., Kim K. Risk analysis and countermeasure for bit-flipping attack in LoRaWAN. Proceedings of the 2017 International Conference on Information Networking (ICOIN).

[21] Yang X. (2017). LoRaWAN: Vulnerability Analysis and Practical Exploitation. Master’s Thesis.

[22] Ingham M., Marchang J., Bhowmik D. (2020). IoT security vulnerabilities and predictive signal jamming attack analysis in LoRaWAN. IET Inf. Secur..

[23] Martinez I., Tanguy P., Nouvel F. (2019). On the performance evaluation of LoRaWAN under Jamming. Proceedings of the 2019 12th IFIP Wireless and Mobile Networking Conference (WMNC).

[24] Ahmar A.U.H., Aras E., Nguyen T.D., Michiels S., Joosen W., Hughes D. (2023). Design of a Robust MAC Protocol for LoRa. ACM Trans. Internet Things.

[25] Hou N., Xia X., Zheng Y. (2023). Jamming of LoRa PHY and Countermeasure. ACM Trans. Sens. Netw..

[26] Huang C.Y., Lin C.W., Cheng R.G., Yang S.J., Sheu S.T. (2019). Experimental Evaluation of Jamming Threat in LoRaWAN. Proceedings of the 2019 IEEE 89th Vehicular Technology Conference (VTC2019-Spring).

[27] José A.N.D.S., Deniau V., Gransart C., Vantroys T., Boé A., Simon E.P. (2022). Susceptibility of LoRa Communications to Intentional Electromagnetic Interference with Different Sweep Periods. Sensors.

[28] Sun Z., Yang H., Liu K., Yin Z., Li Z., Xu W. (2022). Recent Advances in LoRa: A Comprehensive Survey. ACM Trans. Sens. Netw..

[29] Martinez I., Nouvel F., Lahoud S., Tanguy P., Helou M.E. (2020). On the Performance Evaluation of LoRaWAN with Re-transmissions under Jamming. Proceedings of the 2020 IEEE Symposium on Computers and Communications (ISCC).

[30] Torres N., Pinto P., Lopes S.I. (2022). Exploiting Physical Layer Vulnerabilities in LoRaWAN-based IoT Networks. Proceedings of the 2022 IEEE 8th World Forum on Internet of Things (WF-IoT).

[31] Ahmar A.U.H., Aras E., Joosen W., Hughes D. (2019). Towards More Scalable and Secure LPWAN Networks Using Cryptographic Frequency Hopping. Proceedings of the 2019 Wireless Days (WD).

[32] Perković T., Rudeš H., Damjanović S., Nakić A. (2021). Low-Cost Implementation of Reactive Jammer on LoRaWAN Network. Electronics.

[33] Ruotsalainen H. Reactive Jamming Detection for LoRaWAN Based on Meta-Data Differencing. Proceedings of the 17th International Conference on Availability, Reliability and Security.

[34] Kalokidou V., Nair M., Beach M.A. (2022). LoRaWAN Performance Evaluation and Resilience under Jamming Attacks. Proceedings of the 2022 Sensor Signal Processing for Defence Conference (SSPD).

[35] Wadatkar P.V., Chaudhari B.S., Zennaro M. (2019). Impact of Interference on LoRaWAN Link Performance. Proceedings of the 2019 5th International Conference On Computing, Communication, Control And Automation (ICCUBEA).

[36] Perkovic T., Siriscevic D. (2020). Low-Cost LoRaWAN Jammer. Proceedings of the 2020 5th International Conference on Smart and Sustainable Technologies (SpliTech).

[37] Danish S.M., Nasir A., Qureshi H.K., Ashfaq A.B., Mumtaz S., Rodriguez J. (2018). Network Intrusion Detection System for Jamming Attack in LoRaWAN Join Procedure. Proceedings of the 2018 IEEE International Conference on Communications (ICC).

[38] Dossa A., Amhoud E.M. (2025). Impact of Reactive Jamming Attacks on LoRaWAN: A Theoretical and Experimental Study. arXiv.

[39] Beltramelli L., Mahmood A., Österberg P., Gidlund M. (2020). LoRa beyond ALOHA: An investigation of alternative random access protocols. IEEE Trans. Ind. Informatics.

[40] Iglesias-Rivera A., Van Glabbeek R., Guerra E.O., Braeken A., Steenhaut K., Cruz-Enriquez H. (2022). Time-slotted spreading factor hopping for mitigating blind spots in LoRa-Based networks. Sensors.

[41] Gaitan N.C. (2021). A long-distance communication architecture for medical devices based on LoRaWAN protocol. Electronics.

[42] LoRa Alliance Technical Committee. https://lora-alliance.org/resource_hub/lorawan-specification-v1-0-3/.

[43] Haque M.A., Saifullah A. Handling Jamming Attacks in a LoRa Network. Proceedings of the IEEE/ACM International Conference on Internet-of-Things Design and Implementation (IoTDI).

[44] Hou N., Xia X., Zheng Y. Jamming of LoRa PHY and Countermeasure. Proceedings of the IEEE INFOCOM 2021—IEEE Conference on Computer Communications.

[45] Corporation S. (2020). LoRaWAN Protocol Expands Network Capacity with New Long Range—Frequency Hopping Spread Spectrum Technology. https://blog.semtech.com/lorawan-protocol-expands-network-capacity-with-new-long-range-frequency-hopping-spread-spectrum-technology.

[46] Bolivar I.M.M. (2021). Jamming on LoRaWAN Networks: From Modelling to Detection. Ph.D. Thesis.

[47] Chehimi M., Awad M.K., Al-Husseini M., Chehab A. (2023). Machine learning-based anti-jamming technique at the physical layer. Concurr. Comput. Pract. Exp..

